# Expanding the scope of *Endocrine Connections*

**DOI:** 10.1530/EC-23-0051

**Published:** 2023-03-17

**Authors:** A J Clark

**Affiliations:** 1 Endocrine Connections Editor-in-Chief, Emeritus Professor of Endocrinology, University of London, London, United Kingdom of Great Britain and Northern Ireland

*Endocrine Connections* was launched a decade ago with the ambition of becoming the leading fully open access journal that aimed to publish cross-cutting themes in and around endocrinology. When I was appointed Editor-in-Chief in 2021, I did not do so with a ‘manifesto’ for new ideas and change as is often the case with new editors, as I believed that the journal that I took over from Josef Koerle was in the right place. Now, as I come towards the end of my term of office, I am suitably well informed about the journal to identify its strengths and weaknesses.

Anybody with any experience of research knows that not all research results in concept-changing discoveries, but that carefully conducted studies that replicate, refute or support current ideas are equally important and need to be disseminated. *Endocrine Connections* is happy to support such endeavours.

Whilst there is clearly an important place for highly specialized journals in publishing some endocrine research, there is equal importance in sharing ideas between sub-disciplines. This need to support cross-cultivation within and around endocrinology was the basis of our founding editor, Jens Sandahl Christiansen’s aim for the journal, and one to which we still adhere.

Having said this, we consider that there are two areas in which we do not cater sufficiently for the discipline and which we now seek to address. These are the areas of Paediatric Endocrinology and Genetics.

In the case of Paediatric Endocrinology, we believe there are many aspects in which endocrine clinicians and scientists have much to gain from the experience of our colleagues who specialize in endocrine physiology and pathology in childhood and we would like to encourage paediatric endocrinologists to consider *Endocrine Connections* for publishing their research. Conversely, we encourage paediatric endocrinologists to include *Endocrine Connections* on their list of regularly scanned research outputs as we believe the broad scope of this journal may provide valuable insights into their own research questions. To support this initiative, Mehul Dattani has joined the editorial board as an Advisory Editor and we will be seeking to raise our profile at paediatric endocrine meetings. In addition to Professor Dattani, we have already appointed one new senior editor and a number of editorial board members in this area, and will expand our editorial board as we attract more paediatric endocrinology papers. Furthermore, we will encourage the submission of review articles that relate to paediatric elements of disease and, as an example of this initiative, we are currently publishing an excellent series of articles on the ‘Late effects of cancer therapy in children’ (https://ec.bioscientifica.com/page/lateeffects/) and later this year will begin publishing a series on ‘New technologies in diabetes therapy’.

Paediatric disorders frequently reflect the role of inherited factors in their pathogenesis, and expanding the paediatric content of the journal will inevitably expand our genetics content. Few would disagree that the last decade has seen breathtaking developments in the area of genetics, and endocrine genetic understanding has been a major beneficiary of these advances – yet no journal offers an obvious home for basic and clinical research in endocrine genetics. Consequently, we will expand our publication coverage in this topic; to this end we have recruited Constantine Stratakis as an Advisory Editor and are seeking to recruit additional editorial board expertise in this area. As an example of our aspirations in this area throughout the last year, we have been publishing the output from the European Reference Network on Endocrine Rare Diseases (https://ec.bioscientifica.com/page/Endo-ERN%20special%20series/endoern-special-collection) which clearly includes several genetically important contributions. In addition, we are currently publishing a number of articles by world-leading authorities on Klinefelter’s syndrome and related sex chromosome aneuploidies.

In summary, I believe that *Endocrine Connections* is in a good place as a well-established, fully open access journal with a consistently stable impact factor which is jointly owned by and directly supporting two of the largest endocrine societies – the European Society for Endocrinology and the Society for Endocrinology. I would like to thank our highly dedicated Senior Editorial Board and Editorial Board for their hard work that has made the journal what it is today.

## Declaration of interest

The author declares that there is no conflict of interest that could be perceived as prejudicing the impartiality of this editorial.

## Funding

This work did not receive any specific grant from any funding agency in the public, commercial, or not-for-profit sector.

